# New products

**DOI:** 10.1155/S1463924690000293

**Published:** 1990


Journal of Automatic Chemistry, Vol. 12, No. 5 (September–October 1990), pp. 221–226

### New products

### Emergency shutdown Eurocards

A range of Protech Eurocard communications interface modules for monitoring emergency shutdown systems has been announced by Rotork Instruments (formerly Protech Instruments). The new modules, the Protech ES-800 series, feature on-line testing and diagnostics for up to 252 functional modules.

Emergency shutdown systems demand high reliability and ES-800 modules have been designed as an automated system-test facility for maximizing integrity. They do not form part of the logic path and therefore do not compromise the shutdown function. Other facilities provided by these units are alarm sequence indication, alarm logging, truth-table editing and host communications for remote data acquisition and for down-loading of the required truth table.

A Protech ES-800 module is 24E wide and incorporates a local user interface for maintenance and diagnostics with the 16-key operator panel coupled to a high contrast four-line, 16-character LCD display.

Normally the display shows 'OK', otherwise the oldest unacknowledged alarm is given, with identification of shelf and slot numbers and type of fault. A list of output faults taking up more than two lines can be scrolled. The test sequence may be started and stopped from the keyboard. After being stopped manually, it will restart automatically following a time lapse.

In automatic test mode, one functional module is tested every 4 s. Fault sequence logging via a printer or host on a 10 ms cycle provides for one status bit from each functional module logged. Fast (100 Kbits/s data collection is provided via the Protech 'SafetyNet' cubicle-level communications systems. Host interfaces are available in various alternative forms – for a PC using an appropriate card, for Protech 'SuperVision' supervisory software (RS232 port – max. 9·6 KBaud) and for 'Modbus' (RS422/RS485 port – max. 38·4 KBaud).

Configuration of Protech ES-800 modules may be carried out using a Protech portable programming tool.

*Details from Rotork Instruments, Protech House, 241 Selbourne Road, Luton, Bedfordshire LU4 8NP, UK. Tel.: 0582 596181; fax: 0582 598808.*

### High-speed centrifuge

Heraeus Equipment has supplied Unilever Research with a high-speed centrifuge, the Varifuge 20RS. Capable of performing all high-speed and general applications, the unit is being used by Unilever's immunology department for a wide range of research investigations.

The Varifuge 20RS, the world's smallest in its class, is an exceptionally versatile refrigerated centrifuge which can be installed at the work-place. It is suitable for a diverse range of applications including blood separation, protein precipitations and cell fractionations.

The centrifuge features a frequency controlled brushless induction motor which virtually eliminates maintenance and improves reliability dramatically. With a maximum capacity of 3 l and g-force of approximately 50 000, the Varifuge 20RS offers state-of-the-art microprocessor control – the operator can pre-select and store 32 centrifugation programs in the unit's memory and the temperature is programmable in a range from −19 °C to 40 °C. All programs are protected in the event of a power failure.

*More information from Heraeus Equipment Ltd, 9 Wates Way, Brentwood, Essex CM15 9TB, UK. Tel.: 0277 231511; fax: 0277 26856.*

### HPLC

In less than 15 min, the Perkin-Elmer HPLC system separates, purifies and quantitates GeneAmp Polymerase Chain reaction (PCR) products obtained with the Cetus DNA Thermal Cycler. The complete system consists of a biocompatible LC-250 binary pump, a biocompatible variable wavelength UV/Vis detector, a biocompatible 7125 injector kit, an HPLC column kit for PCR analysis and a column fittings kit.

Once amplification is complete, the GeneAmp PCR product can be injected straight into the HPLC system. This is the fastest method of post-PCR analysis, since no preparation steps are required. A chromatogram with sample concentrations indicated for each peak and a complete quantitation report are automatically printed.

*For further information contact Perkin-Elmer Ltd, Maxwell Road, Beaconsfield, Buckinghamshire, UK. Tel.: 0494 676161; fax 0494 678324.*

### Chromatography customization

Philips Analytical Chromatography has expanded its customization service to clients worldwide from its headquarters in Cambridge. Gas and liquid chromatography analyser systems feature advanced multi-column technology for rapid, repeatable performance. Most systems are fully application engineered and supplied with annotated test chromatograms from clients' samples, including customized sample reporting and statistics. The analysers offer leak-proof sampling and column switching valves for automated and process control systems. Advanced data handling on microcomputers ensures validity of results for Good Laboratory Practice in an IBM-compatible environment.

Analyser systems are available on all chromatography instrument ranges. From routine to research grade models, instruments can be rapidly converted to dedicated applications.

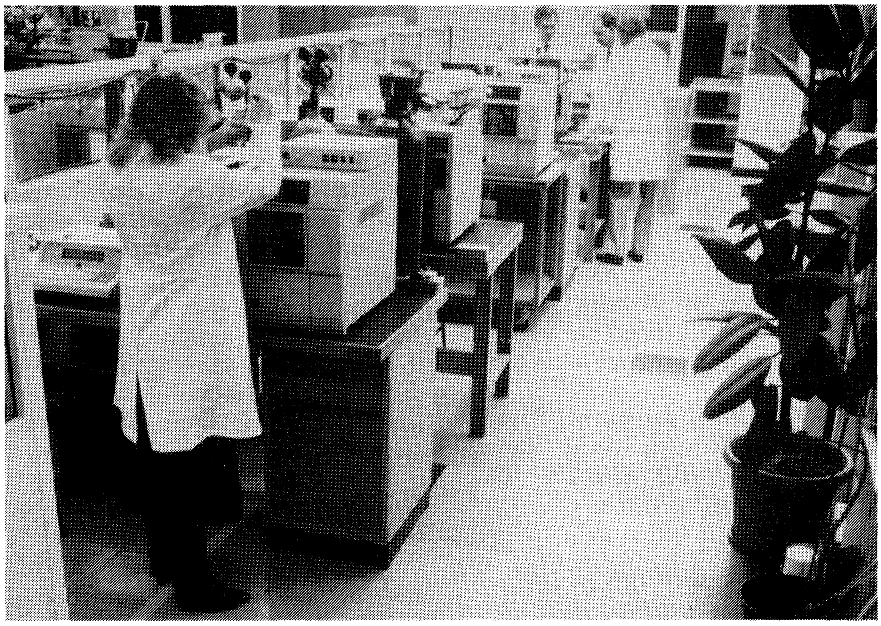

*Chromatography customization on-line at Philips Analytical.*

*Details from Philips Scientific, Analytical Division, York Street, Cambridge CB1 2PX, UK. Tel.: 0223 358866; fax: 0223 312764.*

### Protein and peptide analysis

V. A. Howe & Company have a four-page brochure for Hamilton PRP-3 HPLC Columns; it includes nine application chromatograms that illustrate the versatility of PRP-3 (Polymeric Reversed Phase) 300 ALT 143 HPLC Columns. The advantages of polymeric HPLC columns include:

Good pH stability (from pH 1 to 13) for protein purity determination at either pH extreme without column degradation.

Good protein recovery because the polymeric support is inert (protein recovery is typically 90% or better).

Longer column life (versus C-18 packings) with PRP-3 because of its stability in a wide variety of mobile phases.

*Copies from V. A. Howe & Company Ltd, Beaumont Close, Banbury, Oxfordshire, UK. Tel.: 0295 252666; fax: 0295 268096.*

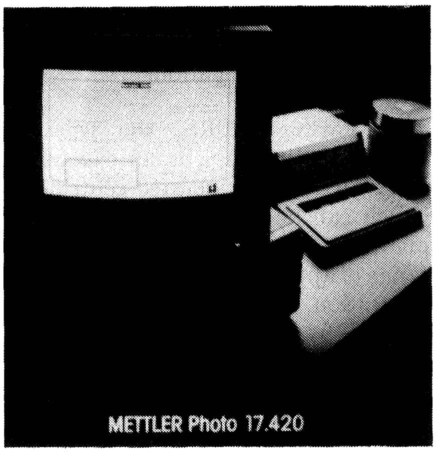

*The FP89 System Software allows a range of quantitative evaluations such as temperature or time of any curve point, peak area integration, crystallinity, melting ranges, onset and endset temperatures, and glass transition temperatures.*

### New software for thermal analysis

The new FP89 System Software from Mettler is a control and evaluation software package that runs under the Microsoft Windows/286 or 386. The advantages of this software include mouse support and pull-down menus.

The FP89 System Software supports the FP85 and FP84HT DSC Measuring Cells of the Mettler FP800HT Thermosystem. The most important performance characteristics of the FP89 evaluation software are: multistage temperature programs (dynamic and isothermal), data management such as storage and sorting of experimental data and test programs, comparisons of experimental curves, zoom function, text entries, as well as interactive qualitative and quantitative evaluations.

The FP89 System Software, with pull-down means, is extremely easy to learn and use. For example, an experimental curve can be displayed and signals evaluated in a matter of seconds. While the various evaluations of a completed measurement are performed, the experiment can be displayed and observed simultaneously in an online window.

*Further information is available from Mettler-Toledo AG, CH-8606 Greifensee, Switzerland.*

### Refrigerated air stream for X-ray diffraction

A mechanically refrigerated system from FTS provides constant flow of dry air or other gas, controllable from −85 °C to +100 °C, for temperature control of X-ray diffraction samples with unattended operation. The gas stream technique provides a useful means to temperature control samples in a manner that does not interfere with visual observation or physical measurements.

*A new brochure describes the features, options, and operation of the system; copies from: FTS Systems, Inc., P.O. Box 158, Route 209, Stone Ridge, New York 12484, USA. Tel.: 914 687 0071; fax: 914 687 7481.*

### Hand-held infrared thermometers

A range of hand-held infrared non-contact thermometers is now available from Comark to complement their existing range of portable instruments. The Thermophil Infra range is especially suitable for the monitoring of temperature during manufacturing processes where products are liable to be damaged if touched.

Measuring over the range −30 to +1300 °C, the 'Thermophil Infra' range offers 'through the lens' sighting combined with a small target area. The target area can be as small as 2 mm square, to provide great accuracy in pinpointing the area to be measured.

Measurements are clear and easy to read, given in 3·5 digit LCD with backlight. Analogue output is also available for connection to recorders or for processing. Readings can be stored, and maximum values also displayed if required.

The Thermophil Infra 4474 measures over the range −30 to 1300 °C; a variety of accessories is available.

*More information from Comark at Artex Avenue, Rustington, Littlehampton, West Sussex BN16 3LN, UK. Tel.: 0903 771911; fax: 093 785773.*

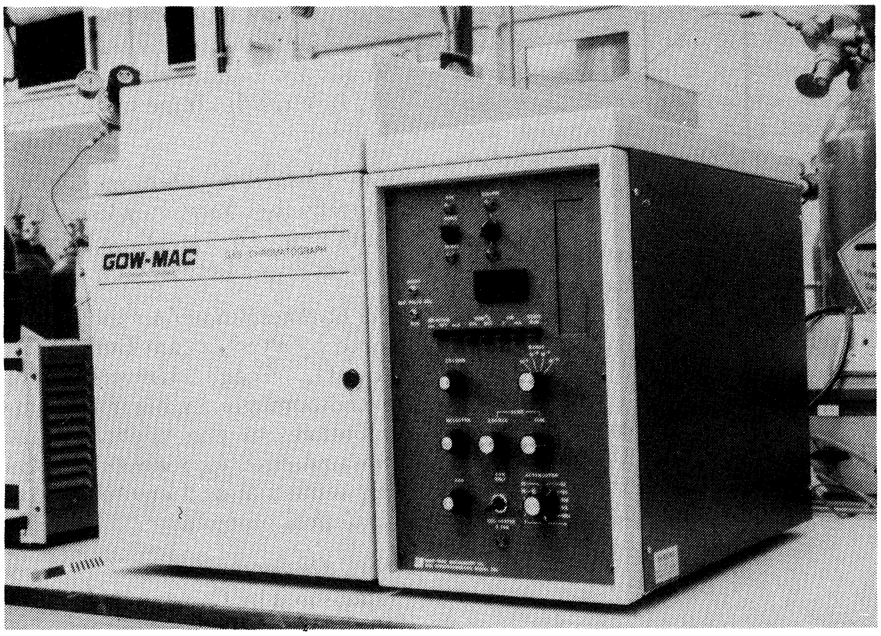

*Gow-Mac Instrument Company's GM 295, a new type of highly sensitive gas chromatograph for the reliable analysis of trace gas impurities in the p.p.b. (parts per billion) range. The analyser utilizes a Discharge Ionization Detector (DID), which detects sample gas components using high energy photoionization. Details from Gow-Mac Instrument Co. (UK) Ltd, Gow-Mac House, PO Box G13, Gillingham, Kent ME7 4HA, UK. Tel.: 0634 575661.*

### BDH gains BS 5750 registration

BDH was awarded comprehensive accreditation under BS 5750: Part 2 for all commercial aspects of its business including reagents, diagnostics, apparatus and industrial chemicals in June 1990. All depots are included in the registration, as well as the site at Chadwell Heath, London and the main manufacturing and administrative sites in Poole, Dorset. BDH is thus the first UK laboratory supplies company to gain the accreditation across the whole of its product range. BDH's registration to the British Standard BS 5750: Part 2: 1987 Quality Systems standard also covers the international standard ISO 9002 and the European standard EN 29002.

*Information about BDH products from BDH on 0202 745520.*

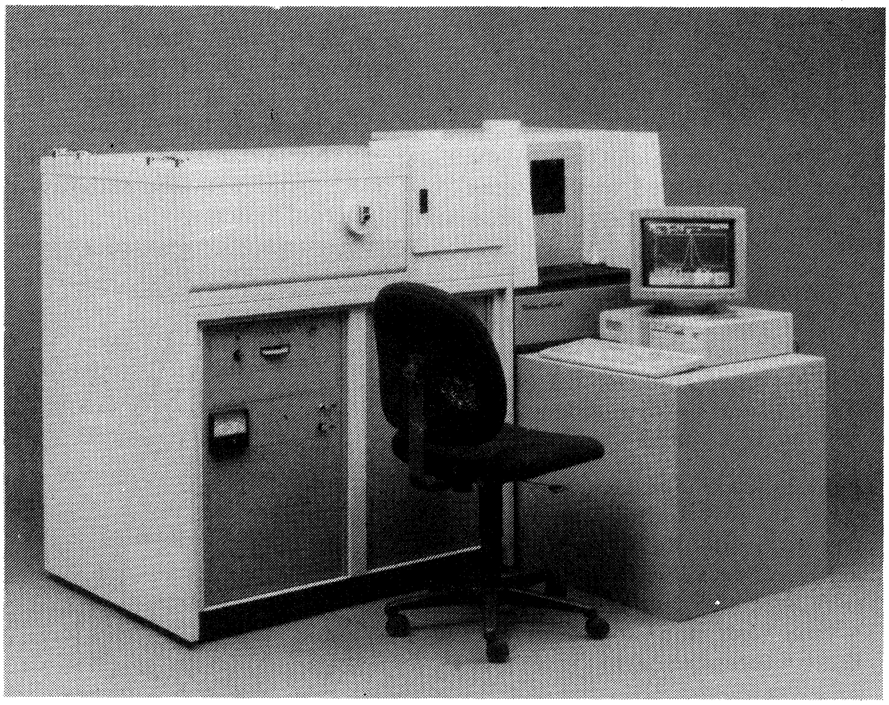

*The ICAP 61E, from Thermo Jarrell Ash, is a simultaneous plasma spectrometer analytical instrument which can be used for the elemental analysis of water, oils, and a variety of other materials. The entire system, including RF power, gas flows, and sample introduction are controlled via an NEC microcomputer. The plasma torch can be operated with low power, low argon flow for routine samples, and high power (up to 2kW) for difficult samples.*

*With our ThermoSPEC Software and a modem, Thermo Jarrell Ash can operate the ICAP 61E remotely via a telephone line to provide applications and troubleshooting assistance and complete control of the entire system from remote location. Details from Thermo Jarrell Ash Corporation, 8E Forge Parkway, Franklin, Massachusetts 02038, USA. Tel.: 508 520 1880; fax: 508 520 1732.*

### Laboratory exhibition for Scotland

A new laboratory exhibition has been launched by the Evan Steadman Communications Group. The Laboratory Scotland Exhibition is to be held on 15 and 16 May 1991 at Glasgow's Scottish Exhibition and

Conference Centre, and will take place simultaneously with the Association of Clinical Biochemists annual conference which is in an adjacent hall. The Association are advisors to the organizers for a sister exhibition, British Laboratory Week.

The exhibition has been launched in response to surveying exhibitors based throughout the UK, who attended recent laboratory exhibitions.

*Details from Evan Steadman Communications Group, The Hub, Emson Close, Saffron Walden, Essex CB10 1HL, UK. Tel.: 0799 26699; fax: 0799 26088.*

The Royal Society of Chemistry's industrially-sponsored awards were presented to the following in June 1990:

*For Analytical Reactions and Analytical Reagents* (sponsored by BDH Ltd), to Professor A. Townshend, University of Hull, for his many contributions to their understanding and application.

*For Analytical Spectroscopy* (sponsored by IMI Ltd), to Dr G. L. Gerrard, BP Research Centre, Sunbury, particularly for his outstanding contribution to the study, development and application of Raman spectroscopy in the chemical industry.

*For Chemical Education* (sponsored by Smith, Kline and French Research Ltd), to Mr D. E. Edwards, Huntington School, York.

*For Chemistry and Electrochemistry of Transition Metals* (sponsored by Inco Europe Ltd), to Dr H. A. O. Hill, University of Oxford.

*For Chemistry of the Noble Metals and their Compounds* (sponsored by Englehard Industries), to Dr W. P. Griffith, Imperial College, London.

*For Combustion Chemistry* (sponsored by Esso Petroleum Company Ltd), to Dr J. F. Griffiths, University of Leeds.

*For Electroanalytical Chemistry* (sponsored by Pye Unicam Ltd), to Dr A. G. Fogg, University of Loughborough, for his many contributions to the application of voltammetric approaches to solving industrial and environmental analytical problems.

*For Macromolecules and Polymers* (sponsored by Courtaulds Ltd), to Dr R. W. Richards, University of Durham.

*For Medicinal Chemistry* (sponsored by The Boots Company PLC), to Dr S. F. Campbell, Pfizer Ltd, for his contributions to medicinal chemistry, particularly in the cardiovascular field.

*For Surface and Colloid Chemistry* (sponsored by Unilever Ltd), to Dr Th. F. Tadros, ICI Agrochemicals, for his work on adsorbed surfactant films and their relationship wtih the stability of dispersed systems.

*For Synthetic Organic Chemistry* (sponsored by CIBA-GEIGY plc), to Professor S. V. Ley, Imperial College, London, for his innovative work in the field of organic synthesis.

*For Theoretical Chemistry* (sponsored by Pergamon Press), to Dr W. G. Richards, Oxford University and Brasenose College, in recognition of the importance of his work in the application of quantum mechanical and statistical mechanical theory, to spectroscopic, chemical, and pharmacological problems.

*For further information contact: The Royal Society of Chemistry, Burlington House, Piccadilly, London W1V 0BN. Tel.: 071 437 8656.*

### HPLC fluorescence detector

The SA 6604 programmable HPLC fluorescence detector from Severn Analytical, offers chromatographers exceptional performance and versatility. Many features of the SA 6604 have been incorporated to aid the optimization of the detection parameters and provide a reliable and sensitive instrument for both research and routine applications.

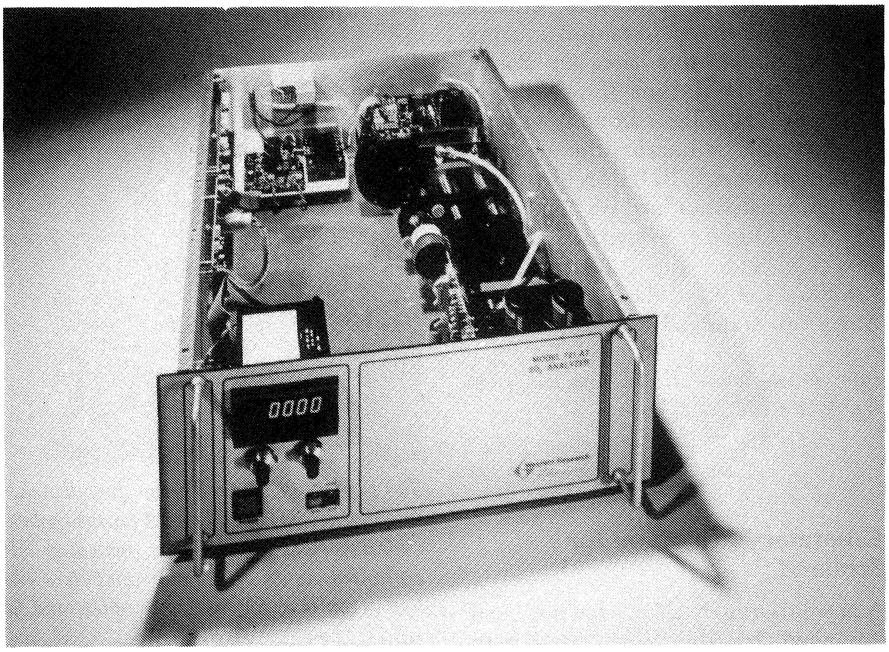

*The 19-in rack-mounting enclosure for the Western Research Model 721AT $SO_2$ analyser, which has been launched into Europe through Ludlam Sysco Ltd, showing the digital display for $SO_2$ concentration. Details from Ludlam Sysco Ltd, Broadway, Market Lavington, Devizes, Wiltshire SN10 5RQ, UK. Tel.: 0380 818411; fax: 0380 812733.*

The SA 6604 is a compact instrument with a clear LCD display which facilitates input of analysis conditions. The ability to program both excitation and emission monochromators allows the analyst to optimize detection in a mixture where the absorption and fluorescence characteristics of the individual compounds are very different, while programmability of range changes during a chromatographic run overcomes the problems of compounds being present at widely differing concentrations, aiding both quantification and presentation.

A scanning option is available which enables the chromatographer to obtain the fluorescence spectra of peaks as they elute from the HPLC column, providing essential information about wavelengths selection and peak purity.

Signal to noise ratio, and hence sensitivity, can be fine-tuned by controlling the flash rate of the Xenon source and by the use of additional static filtering. The Xenon lamp is also provided with a power switch, independent from the main instrument, and so can be used as a chemiluminescence or phosphorescence detector. The SA 6604 allows easy access to the flow cell and lamp housing, making routine maintenance simple and convenient.

*Reader enquiries to Severn Analytical Ltd, Unit 2B, St Francis Way, Shefford Industrial Park, Shefford, Bedfordshire, UK.*

### GeneAmp RNA PCR kit

The GeneAmp RNA PCR Kit from Perkin-Elmer contains all the components necessary for transcription of RNA to cDNA and amplification of cDNA using the GeneAmp Polymerase Chain Reaction (PCR) process. Researchers can use the kit to generate a cDNA product in excess of 2Kb from all types of RNA.

The new kit is applicable to a wide range of studies, including amplification of cDNA derived from all different types of RNA (mRNA, tRNA, rRNA, total RNA, viruses) and detection of specific RNA species present at low copy number.

All reagents have been premixed and matched in ratios optimized for efficient reverse transcription of RNA and subsequent amplification of the resulting cDNA products using the GeneAmp PCR process. Both reactions are performed in a single tube using the same buffer. The sensitivity of RNA detection provided by combining the reverse transcription and GeneAmp PCR amplification processes is unmatched by other available techniques used for analysis of RNA.

The kit incorporates the components of the new GeneAmp PCR Core Reagents, and includes a positive control RNA template transcribed from the plasmid pAW 109, control primers that flank an IL-1 alpha sequence in the control RNA, random hexamers, reverse transcriptase, RNase inhibitor, dATP, dTTP, dCTP and dGTP. Sufficient positive control RNA is provided to perform 25 reverse transcription/amplification reactions starting with $10^4$ copies per reaction.

Positive/negative information on multiple samples can be simultaneously obtained in a matter of hours. The GeneAmp RNA PCR Kit not only provides sensitive detection and analysis of gene expression at the RNA level, but it can also be used to generate cDNA for cloning purposes. The kit eliminates the need for large-scale RNA preparation.

*For further information on the PCR process, contact Perkin-Elmer Limited, Maxwell Road, Beaconsfield, Buckinghamshire HP9 1QA, UK. Tel.: 0494 676161; fax: 0494 678324.*

### Price reduction

Chromacol, a major supplier of chromatography vials, caps and seals, has announced a 30% reduction in the prices of its popular 4-SV and 4-SV(A) vials. The new prices came into effect on 15 July 1990.

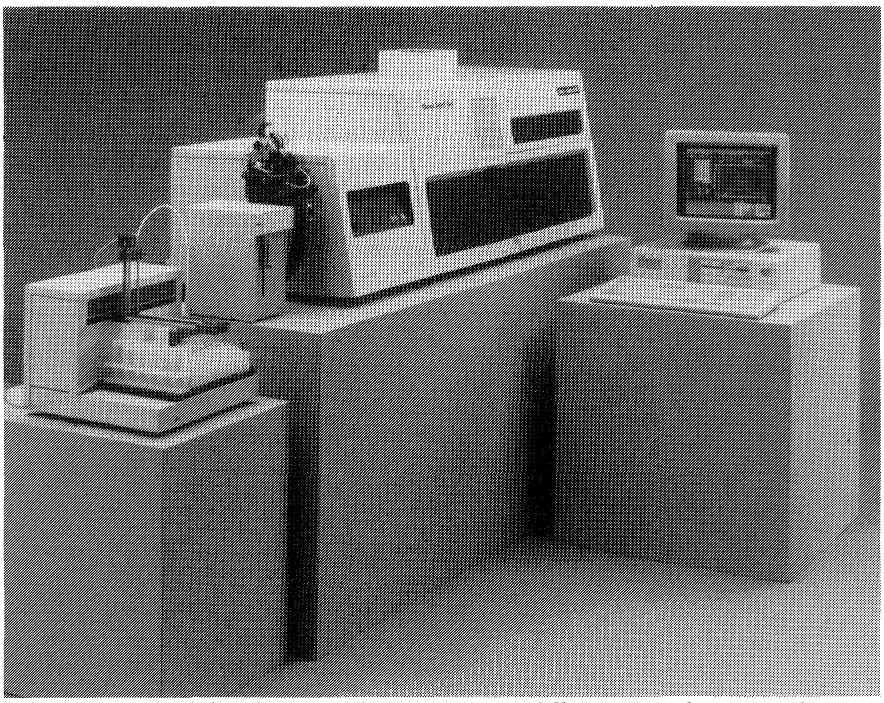

*Thermo Jarrell Ash's Smith-Hieftje 8000, is a fully automated atomic absorption spectrometer compatible with both flame and furnace atomization. Automated for both ease of use and unattended operation, the Smith-Hieftje 8000 is computer controlled through a NEC PowerMate 286 Plus computer with ThermoSPEC® Software. All spectrometer parameters including spectral bandpass, wavelength, lamp selection, lamp currents, background correction (deuterium or Smith-Hieftje), detector voltages as well as furnace parameters are automatically set from a stored program. In addition to the capability of measuring up to eight elements in a single unattended run, the Smith-Hiefje 8000 has the additional capability of measuring two elements simultaneously by furnace and four elements simultaneously by flame AAS. Details from Thermo Jarrell Ash Corporation, 8E Forge Parkway, Franklin, Massachusetts 02038, USA. Tel.: 508 520 1880; fax 508 520 1732.*

These are the standard vials used with the Waters Wisp$^{TM}$ 48 vial tray but are also suitable for use with other autosamplers from Kontron, Shimadzu and Gynkotek.

The vials still retain Chromacol's high quality, are made from borosilicate glass, the cost savings coming mainly from larger production runs.

*Further information from Chromacol Ltd, Glen Ross House, Summers Row, London N12 0LD, UK. Tel.: 081 368 7666; fax: 081 361 4698.*

### BP Chemicals sells part of speciality acetate business

BP Chemicals has announced (3 July 1990) the sale to MTM PLC of two of the four main product areas which comprise its speciality acetates business at Carshalton, Surrey. This sale forms part of the phased closure of BP Chemicals Carshalton site announced in February 1990. As a result of the sale MTM will acquire the goodwill, technology and customer lists for ethyl hexyl acetate, ethylene glycol diacetate (EGDA) and blends of EGDA; and the minor products ethylene diglycol acetate, propylene glycol diacetate and the non-perfumery grade of nonyl acetate. These products are used in a variety of industrial processes in addition to specialist applications in the foundry and tanning sectors. Associated manufacturing plant and equipment will also be purchased by MTM, and will be transferred to the company's Speciality Chemicals sites at Leek in Staffordshire and Sandbach, Cheshire.

*Details from BP Chemicals Ltd, Belgrave House, 76 Buckingham Palace Road, London SW1W 0SU. Tel.: 071 581 6651; fax: 071 581 6475.*

### Programmable, precision dispensing robot

Iwashita's Autoshooter-7 ASC 7000 is now available in the UK from Hakuto International. Designed for use in industrial processes, the system is well suited to many dispensing applications. Capable of performing smooth, fast metering of a wide range of liquids in linear or circular modes, the ASC 7000 can be easily programmed for fully automated operation.

The standard system consists of a robot controller and *x–y* stage, to which a wide variety of dispenser heads can be fitted. Modular construction allows customization to many different applications. For example a table-top configuration is available to create a stand-alone system, or the controller and *x–y* stage can be separated for incorporation into other equipment or production lines.

The robot controller is 1300 step programmable, with a facility to save the input data to disk. Smooth dispensing operations in linear, circular, copy and surface coating can be performed via PTP and CP control methods.

A number of different dispense controllers are also available. This enables selection of shot time ranges from 0·001 to 99·99 s at shot volumes as low as 0·0001 ml. All dispense controllers incorporate a vacuum transducer and are capable of handling a wide range of liquid viscosities. Additionally, the programmable controller can store up to eight different shot times, input by the 10 key pad or by teaching.

The versatility and ease of use of this system make it attractive for many production line dispensing operations, where accurate control, combined with speed and a high degree of automation are essential considerations.

*Details from Hakuto International Eleanor House, 33–35 Eleanor Cross Road, Waltham Cross, Hertfordshire EN8 7LF, UK. Tel.: 0992 769090; fax: 0992 763300.*

